# Differential activity of wheat antioxidant defense system and alterations in the accumulation of osmolytes at different developmental stages as influenced by marigold (*Tagetes erecta* L.) leachates

**DOI:** 10.3389/fpls.2022.1001394

**Published:** 2022-12-01

**Authors:** Rayees Ahmad Mir, Surendra Argal, Mohammad Abass Ahanger, Keshav Singh Jatav, Rajiv Mohan Agarwal

**Affiliations:** ^1^ School of Studies in Botany, Jiwaji University, Gwalior, India; ^2^ Department of Botany, Government Chhatrasal College, Shivpuri, India

**Keywords:** allelopathy, antioxidant system, osmolytes, *Tagetes erecta* L., wheat

## Abstract

Experiments were conducted to evaluate the effectivity of *Tagetes erecta* L. leachates on various growth, physiological, and biochemical parameters of wheat at different stages of growth. Results suggested that *Triticum aestivum* L. seedlings/plants when exposed to higher concentrations of marigold leachates (10%, 20%, and 30% w/v of fresh parts and 5% and 10% w/v of dry parts) exhibited enhanced lipid peroxidation along with an increase in the activity of protease and phenylalanine ammonia lyase. Treatment with higher concentrations of leachates of fresh (30% w/v) and dry (10% w/v) *T. erecta* upregulated the activity of superoxide dismutase, catalase, ascorbate peroxidase, guaiacol peroxidase, glutathione S-transferase, and glutathione reductase and also increased the non-enzymatic components of antioxidant defense such as glutathione, ascorbic acid, and total phenols along with osmotic constituents comprising free proline, free sugars, and free amino acids in wheat. The growth and yield attributes of wheat exhibited a slight increase at treatments with lower concentrations (1% w/v) of dry leachates, whereas a decrease was recorded at higher concentrations (10% w/v). In general, treatments with flower leachates (higher concentrations) showed greater influence as compared with those with leaf leachates. Identification and understanding the mechanism of function of allelochemicals in these leachates may pave a way for further experimentation on *Tagetes erecta* L crop while it is cultivated and decomposed in the field.

## Introduction

In nature, plants synthesize secondary metabolites/allelochemicals which may be present in all types of plants and tissues and may not be involved in their primary metabolism. These compounds vary with different species, seasons, and age of plants (Tomar et al., 2015; Mir et al., 2018; Mir et al., 2021) and are released into the environment by a variety of mechanisms including decomposition of fallen parts, volatilization, root exudation, and other mechanisms/sources. The action/effect of allelochemicals on their release into the environment have been observed by different workers (Mishra and Nautiyal, 2012; Gallego et al., 2020). The action of these allelocompounds varies with the target plants, affecting various biochemical processes, which results in modification of diverse physiological functions (Gniazdowska and Bogatek, 2005). Survival of plants under such circumstances depends on the plant defense to allelochemical detoxification (Gniazdowska and Bogatek, 2005; Mir et al., 2021).

Environmental stresses biotically/abiotically affect physiological and biochemical processes in plants like photosynthesis, plant water relations, and membrane integrity due to the excess production of reactive oxygen species (ROS) which results in metabolic dysfunction and leakage of cellular components (Gill and Tuteja, 2010; Ahanger et al., 2017b). The immediate production of different osmoprotectants and activation or modification of antioxidant defense system play an important role under such conditions to keep cells away from immediate cellular damage (Jamil et al., 2005; Mir et al., 2020). Accumulation of compatible osmolytes such as proline, amino acids, and sugars (Ahanger et al., 2017a); antioxidant components like superoxide dismutase (SOD), catalase (CAT), ascorbate peroxidase (APX), glutathione reductase (GR), glutathione-*S*-transferase (GST), ascorbic acid (AsA), and glutathione (GSH); and phenolic compounds protect plants from oxidative damage by scavenging the excess ROS and maintains the cellular homeostasis (Gill and Tuteja, 2010; Ahanger et al., 2017b).

Allelochemicals after getting released into the environment become stressful only when they affect the growth and development of receptor plants in the surrounding, i.e., phytotoxicity (Cruz-Ortega et al., 2007). The different cultivars of crops with allelopathic activity can be grown through intercropping with other crops/as cover crop/crop rotation, by use of mulches, etc., in order to suppress weeds under field conditions (Jabran et al., 2015). Various crops reportedly show allelopathic effects on associated weeds of wheat, e.g., *Sorghum* and sunflower (Kandhro et al., 2015) and *Brassica*, *Sorghum*, and sunflower (Awan et al., 2012; Arif et al., 2015). In the northern plains of India including the states of Punjab, Haryana, Uttar Pradesh, Rajasthan, Madhya Pradesh, and Bihar, wheat is a dominant crop, and on the world scenario India is the fourth major wheat-producing country (Kulshrestha, 1985). It plays an important role in fulfilling food and energy requirements and in the livelihood of farmers. Wheat is the major source of starch and energy providing significant quantity of components which are useful for health like proteins, vitamins, dietary fiber, and important phytochemicals which help to reduce the risk of cardiovascular diseases, type 2 diabetes, etc. (Shewry and Hey, 2015).

*Tagetes erecta* L. is a commonly grown species mostly for its bloom and natural dye extraction (Raghava, 1998) exhibiting nematicidal, fungicidal, and insecticidal activities (Xu et al., 2011) and has been used as a cover crop to protect plant-parasite nematodes, inhibition of growth of microorganisms, and pesticides (Silveira et al., 2009; Hooks et al., 2010). The important secondary metabolites which *Tagetes erecta* L. contains include terpenes, essential oils, flavonoids, carotenoids, and polyphenols (Xu et al., 2011). Among different flowering crops, *Tagetes* is an important traditional flower crop raised in India having adaptability to diverse climatic conditions (Raghava et al., 2013), receiving great attention in scientific research because of its tremendous commercial usage (Mir et al., 2019). Aqueous leaf and stem extracts of *Tagetes erecta* inhibited its own seed germination and seedling growth, i.e., showing autotoxicity (Kaul, 2000). Inhibition of radish was more compared with lettuce when exposed to aqueous extracts of *Tagetes* species (Kaul and Bedi, 1995). In India, flowers of marigold (*Tagetes erecta* L.) are largely used by people on various social and religious occasions such as decoration of Mandaps/Pooja places, temples, marriage ceremonies, and houses and cars during festivals, and after use these flowers may be disposed off in agricultural fields, which may affect the crops growing therein. It is with this background that the present work has been undertaken to evaluate the effect of marigold leachates on wheat.

## Material and methods

### Plant material and experimental site

Certified seeds of marigold (*Tagetes erecta* L.) cultivar Pusa Basanti Gainda (PBG) developed by the Indian Agriculture Research Institute, New Delhi, were obtained from the Horticulture and Landscaping Division, Indian Agriculture Research Institute, Pusa, New Delhi, India, and the cultivation was done in the Botanical Garden of School of Studies in Botany, Jiwaji University, Gwalior. The collection of samples for the preparation of leachates was done using these cultivated plants. Similarly, the grains of test crop wheat *Triticum aestivum* L. variety LOK-1 which is a commonly cultivated variety in the state of Madhya Pradesh were procured from Krishi Vigyan Kendra, Rajmata Vijayaraje Scindia Krishi Vishwa Vidyalaya, Gwalior, and were raised under both laboratory and field conditions in the School of Studies in Botany, Jiwaji University, Gwalior, for evaluating the effects of marigold leachates.

### Preparation of leachates

#### Leachates of fresh parts

Preparation of fresh leachates was done in accordance with Tomar and Agarwal (2013) in which clean fresh parts (leaves and flowers) of marigold cv. PBG were cut into small pieces, weighed, and soaked in distilled water at room temperature for 48 h. The leachates were squeezed after 48 h with the help of double-layered cheese cloth followed by centrifugation. The 30% leachates of fresh leaves (FLL) and fresh flowers (FFL) were made from 30 g of initially weighed plant sample, and the lower concentrations (5%, 10%, and 20%) were prepared by further diluting these solutions with distilled water which were then finally stored at 4°C for bioassay.

#### Leachates of dry parts

Dry leachates were also prepared in a similar way (Tomar and Agarwal, 2013). Briefly, an oven-dried 10-g (powdered) plant sample was soaked in 100 ml distilled water at room temperature for 48 h, filtered, and centrifuged after 48 h of soaking. A supernatant was made up to 100 ml with distilled water which is considered as 10% leachates of dry leaves (DLL) and dry flowers (DFL), and the lower concentrations (1% and 5%) were prepared by diluting these solutions and were subsequently stored at 4°C for bioassay experiments. Laboratory and pot experiments (using soil and sand) were conducted to evaluate the impact of leachates of fresh/dry leaves and flowers of marigold on wheat.

### Experimental setup/design

Three types of experiments conducted are as follows.

#### Laboratory experiments

For laboratory experiments, 10 surface-sterilized wheat grains using mercuric chloride (0.01%) solution followed by thorough washing with distilled water were put in each petri plate, lined with Whatman no. 1 filter paper. Leachates of dry plant material (leaves/flowers) were applied to petri plates which served as different treatments, and the replicates without leachates supplied with the same quantity of distilled water served as control. The analysis of different antioxidant and osmotic components in seedlings grown under different treatments was carried out on the 7th day of seed wetting.

#### Pot experiments using sand substrate

Filtered sand using a sieve (2.5–5.0 mm) was washed thoroughly four to five times by tap water and was kept for 24 h in 18% HCl (Hewitt, 1966) followed by repeated washings with distilled water till the whole acid is washed away. Sterilized (LOK-1) wheat grains were sown in sand-filled plastic pots having a diameter of 18 cm. After germination, 200 ml of full-strength Hoagland’s solution was supplied to each pot, and after 10 days of germination, wheat seedlings grown in pots were subjected to different leachate treatments:

- Different concentrations (5%, 10%, 20%, 30%) of leachates (made in full-strength Hoagland’s solution) of fresh leaves and flowers of *Tagetes erecta* L. cv. PBG in one set
- Different concentrations (1%, 5%, 10%) of leachates (made in full-strength Hoagland’s solution) of dry leaves and flowers of *Tagetes erecta* L. cv. PBG in another set

Wheat seedlings raised in pots were fed with 200 ml of fresh/dry leachates of different concentrations on every alternate day, and the same quantity of Hoagland’s solution without leachates was supplied to control. The analysis of different growth and antioxidant attributes/components of plants grown under different treatments was carried out after 25 days of seed wetting. Screening experiments on 25 DAS-old plants in sand cultures using both fresh and dry leachates of leaves and flowers of *Tagetes erecta* L. cv. PBG made it obvious that dry leachates have greater impact as compared with fresh leachates. Therefore, for further experimentation both in laboratory and in field, only dry leachates were used.

#### Pot experiments using soil substrate

Pot experiments were conducted in polythene bags having an area of 962 cm^2^. Each time/season, polythene bags were refilled with fresh soil. Wheat plants raised in pots/polythene bags treated with dry leachates of marigold parts were arranged in randomized block design. The plants so raised fall in the following categories, and at least four independent pots/polythene bags were maintained for each treatment:

- Wheat plants without leachates, irrigated with normal water, were considered as control.
- Wheat plants treated with (1%, 5%, and 10%) leachates of dry leaves and flowers of marigold cultivar PBG.

After 15 days of seed sowing, 200 ml leachates of dry parts was supplied to each pot after. The plants so raised were put to analysis as outlined below at the vegetative (pre-flowering) and flowering stages. Morphological parameters like plant height, spike length, and number of spikelets per spike were recorded at the flowering stage, whereas yield parameters like seed weight were recorded after the crop harvest.

### Measurement of lipid peroxidation and protease activity

Lipid peroxidation was measured according to the method of Heath and Packer (1998). Briefly, 300 mg of fresh plant tissue was extracted in 0.1% TCA followed by centrifugation at 10,000 g. Thereafter, 1 ml supernatant was reacted with 4 ml (20%) TCA containing 0.5% thiobarbituric acid (TBA) in a boiling water bath. Samples were again centrifuged for 15 min at 10,000 g, and the supernatant was read at 532 and 600 nm. MDA content was calculated using an extinction coefficient (ϵ) of 155 mM^-1^ cm^-1^.

Protease (EC 3.4.21.40) activity was determined according to Green and Neurath (1954). To the 1-ml supernatant was added 1 ml casein followed by incubation at 40°C for 1 h. Samples were removed and brought to room temperature followed by the addition of chilled TCA (20%) and kept in a freezer for 2–3 h. Samples were again centrifuged at 5000g for 15 min, and the supernatant was made alkaline by adding 1 mL NaOH (1.5 N) and the volume made to 5 ml using buffer. A 1.0-ml alkaline supernatant was mixed with 5 ml reagent C (prepared as in estimation of protein) and left for 5 min followed by addition of 1.0 ml Folin–Ciocalteu’s reagent and allowed to stand for 30 min, and samples were read at 660 nm. Activity was expressed as µg tyrosine released mg^-1^ protein.

### Assay of antioxidant enzymes

For assay of superoxide dismutase (SOD), catalase (CAT), ascorbate peroxidase (APX), guaiacol peroxidase (GPX), glutathione S-transferase (GST), and glutathione reductase (GR), 100 mg of fresh plant tissue was homogenized using mortar and pestle in a chilled 2.0-ml extraction mixture (100 mM potassium phosphate buffer, pH 7.8) containing PVP (0.1%) and EDTA (0.5 mM) followed by centrifugation at 12,000 g. The supernatant so obtained was utilized for enzyme assay, and the whole process was carried out under cold conditions (4°C). Extraction buffer was supplemented with 2 mM ascorbate for the extraction of APX. SOD (EC 1.15.1.1) activity was estimated as the ability of enzyme extract to inhibit the photochemical reduction of NBT (Dhindsa et al., 1981). CAT (EC 1.11.1.6) activity was measured according to Aebi (1984), and the change in optical density was recorded at 240 nm for 2 min and expressed as EU mg^-1^ protein. For APX (EC 1.11.1.1) assay, H_2_O_2_-dependent oxidation of ascorbate was followed by change in the absorbance at 290 nm (Nakano and Asada, 1981) and expressed as EU mg^-1^ protein. GPX (EC 1.11.11.11) activity was determined following Chance and Maehly (1955), and an increase in absorbance was observed at 470 nm for 2 min and expressed as EU mg^-1^ protein. GST activity was determined spectrophotometrically following Habig et al. (1974) The reaction was initiated by the addition of 1-chloro-2,4-dinitrobenzene (CDNB), and the change in absorbance at 340 nm was monitored for 2 min. GR (EC 1.6.4.2) activity was determined following Foyer and Halliwell (1976), and glutathione dependent oxidation of NADPH was measured at 340 nm for 3 min and was expressed as EU mg^-1^ protein.

### Estimation of non-enzymatic antioxidants

Reduced glutathione GSH was determined by the method of Moron et al. (1979). A fresh plant sample (0.5 g) was homogenized in 5% TCA (2.5 ml) and centrifuged at 10,000g for 10 min. A volume of 100 µl was made up to 1.0 ml by adding 0.2 M sodium phosphate buffer (pH 8.0) followed by the addition of 2.0 ml freshly prepared 5,5-dithiobis nitrobenzoic acid, and absorbance was measured spectrophotometrically at 412 nm. Computation was done using a standard curve of GSH.

For estimation of ascorbic acid, the method of Omayl et al. (1979) was followed. The 100-mg plant tissue was extracted in 10% TCA and centrifuged at 3,500g for 20 min. A 0.5-ml supernatant mixed with 1 ml DTC reagent [prepared by dissolving 3 g of DNPH, 0.4 g of thiourea, and 0.05 g CuSO_4_ in 100 ml 9N H_2_SO_4_] was added. After incubating for 3 h at 37°C, 0.750 ml of ice-cold H_2_SO_4_ (65%) was added. After 30 min, absorbance was read at 520 nm. A standard curve of ascorbic acid was used for calculation and expressed as mg g^-1^ weight.

#### Total phenols

Total phenols were estimated following the method of Malick and Singh (1980). Phenols react with phosphomolybdic acid in Folin–Ciocalteau’s reagent in alkaline medium and produce a blue-colored complex. The concentration of phenols was expressed in mg g^-1^ fresh weight, equivalent to catechol.

Estimation of tannins was done according to the method of Swain and Hills (1959). To a 0.1-ml extract, 1 ml Folin–Denis reagent was added followed by addition of 2 ml sodium bicarbonate (35%) and the resultant was incubated for 45 min in the dark at room temperature. Optical density was recorded at 700 nm, and the computation was done by using a tannic acid standard curve.

### Assay of phenylalanine ammonia-lyase

PAL was extracted from fresh tissue in 0.1 M borate buffer followed by centrifugation at 12,000g. An assay mixture containing borate buffer (pH 8.8), 0.2 ml enzyme, and 1 ml phenylalanine (20 mM) was incubated at 30°C followed by addition of TCA (1 M). Optical density was recorded at 290 nm, and the activity was expressed as nmol trans-cinnamic acid min^-1^ mg^-1^ protein Zucker (1965).

### Total antioxidant activity

The total antioxidant activity was estimated in accordance with Shimada et al. (1992). Tissue was extracted in methanol containing 0.1% HCl and centrifuged at 10,000g. The 0.1-ml supernatant was mixed with 2.0 ml freshly prepared 0.1 mM DPPH (1,1-diphenyl-2-picrylhydrazyl). After 30 min, absorbance was recorded at 517 nm and percent DPPH scavenging activity was calculated using the following equation:


$$\text{DPPH scavenging ability}=\left[1-\frac{{\text{A}}_{\text{i}}{\text{-A}}_{\text{j}}}{{\text{A}}_{\text{c}}}\right]\times 100$$

where

A_i_ = absorbance of extract + DPPH,
A_j_ = absorbance of extract + methanol, and
A_c_ = absorbance of DPPH + methanol.

### Estimation of osmolytes

Total free sugars were estimated using the anthrone method (Fong et al., 1953; Jain and Guruprasad, 1989), and glucose was used as standard. Free amino acids were estimated according to the method outlined in Sadasivam and Manickam (2004) using glycine as standard. Free proline estimation was done following Bates et al. (1973). Extraction was carried out in 3% sulfosalicylic acid, and the supernatant was reacted with acid ninhydrin reagent at 100°C for 1 h. Reaction was terminated in ice followed by the separation of free proline using toluene, and the optical density was recorded at 520 nm. Computation was done using proline standards.

Relative water content Weatherley’s (1950) method was used to measure the relative water content (RWC) in fully expanded wheat leaves.

### Statistical analysis

The appropriate standard error ( ± SE) was calculated using at least four independent replicates. Histograms were prepared using SigmaPlot 11.0 software. Data were statistically analyzed using analysis of variance (ANOVA) with the help of Statistix 8.1 software, and the treatment means were analyzed by using the LSD test at p< 0.05.

## Results

### Lipid peroxidation and protease activity in wheat seedlings/plants

**Figure 1:**
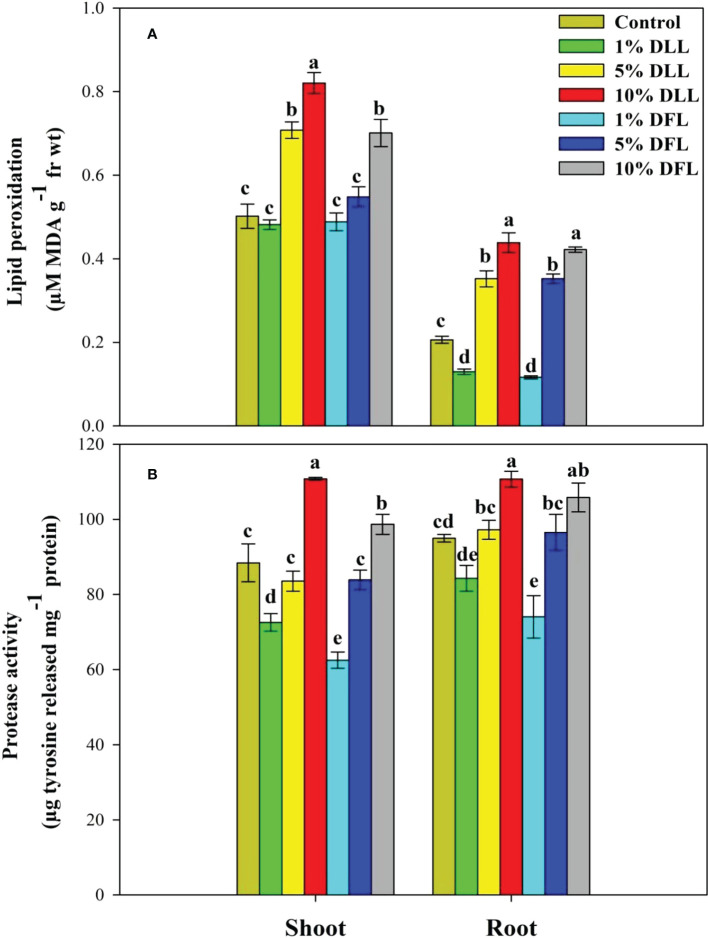
**(A)** Lipid peroxidation (µM MDA g^-1^ fr wt) and **(B)** protease (µg tyrosine released mg^-1^ protein) activity in *Triticum aestivum* L. seedlings (7 DAS) treated with leachates of dry leaves and flowers of *Tagetes erecta* L. Data followed by the same letters are not significantly different at p< 0.05.

**Figure 2:**
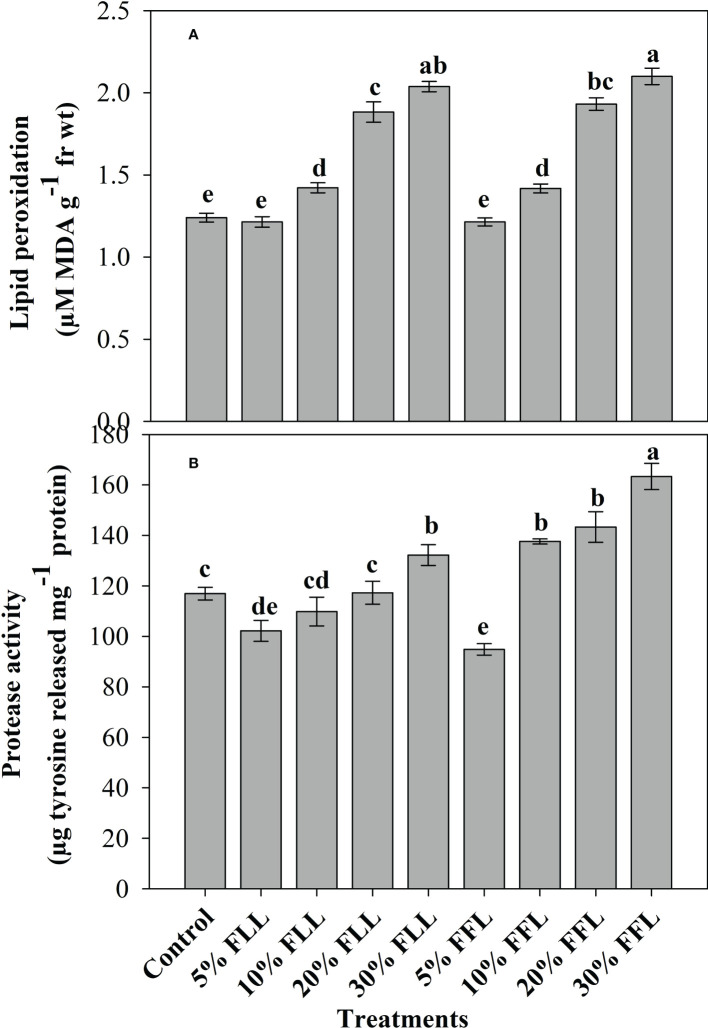
**(A)** Lipid peroxidation (µM MDA g^-1^ fr wt) and **(B)** protease (µg tyrosine released mg^-1^ protein) activity in leaves of *Triticum aestivum* L. plants raised using sand substrate (25 DAS) treated with leachates of fresh leaves and flowers of *Tagetes erecta* L. Data followed by the same letters are not significantly different at p< 0.05.

**Figure 3:**
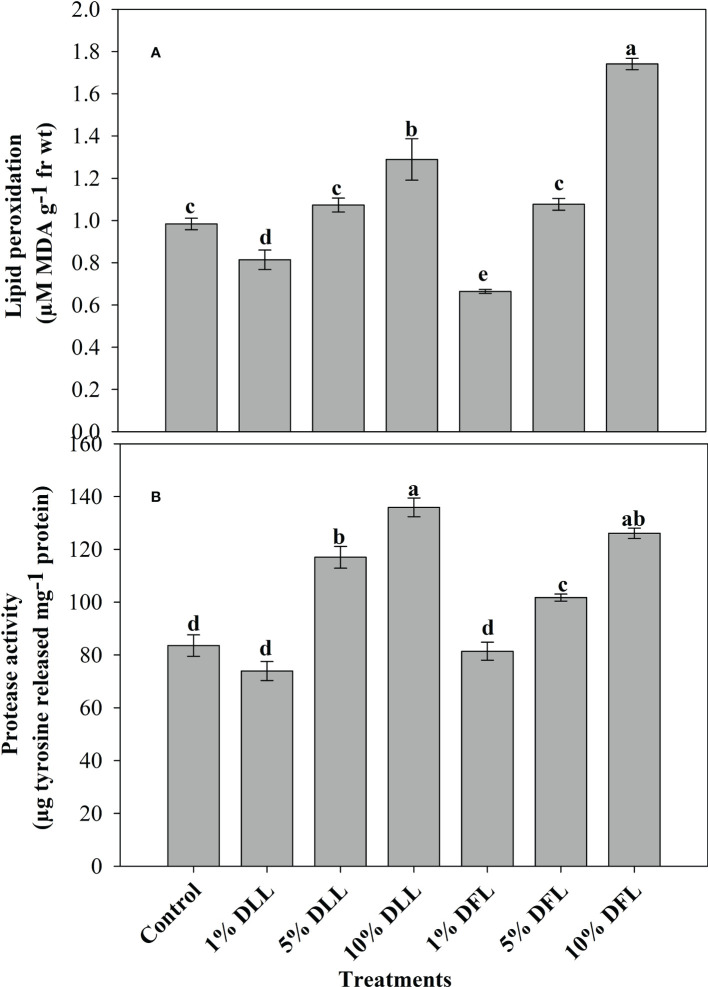
**(A)** Lipid peroxidation (µM MDA g^-1^ fr wt) and **(B)** protease (µg tyrosine released mg^-1^ protein) activity in leaves of *Triticum aestivum* L. plants raised using sand substrate (25 DAS) treated with leachates of dry leaves and flowers of *Tagetes erecta* L. Data followed by the same letters are not significantly different at p< 0.05.

**Figure 4:**
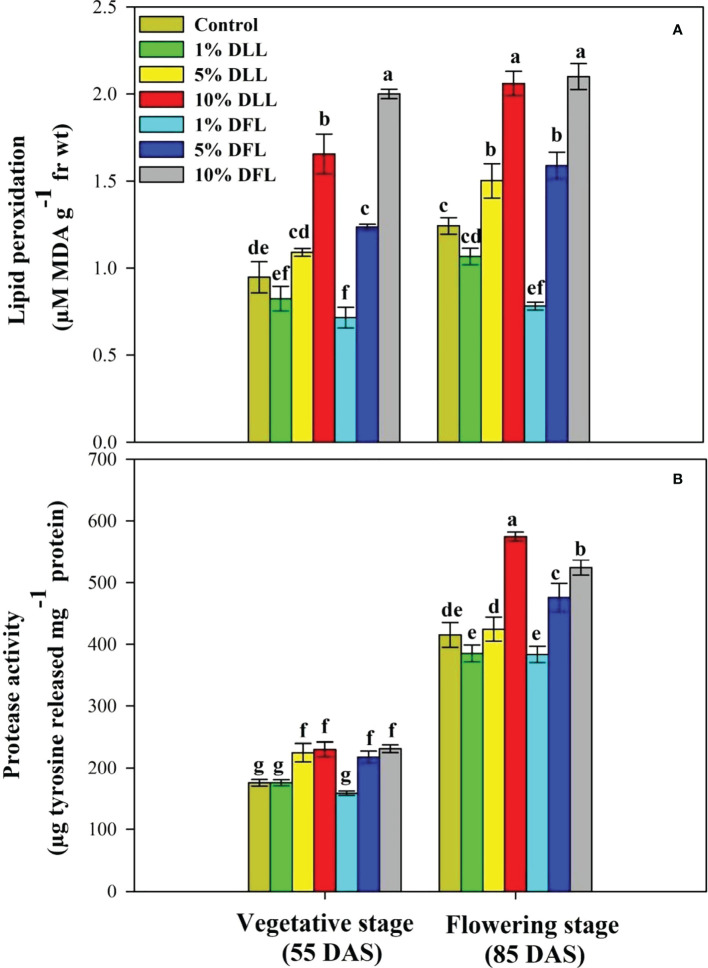
**(A)** Lipid peroxidation (µM MDA g^-1^ fr wt) and **(B)** protease (µg tyrosine released mg^-1^ protein) activity in flag leaves of *Triticum aestivum* L. raised using soil substrate at different developmental stages treated with leachates of dry leaves and flowers of *Tagetes erecta* L. Data followed by the same letters are not significantly different at p< 0.05.

*Triticum aestivum* L. seedlings/plants exposed to higher concentrations of leachates 10%, 20%, and 30% w/v of fresh parts and 5% and 10% w/v of dry parts of marigold showed an increase in lipid peroxidation and protease activity (**Figures 1**–**4**). Wheat plants raised using sand substrates under field conditions receiving 30% w/v leachates of fresh parts showed maximum enhancement in lipid peroxidation and protease activity; moreover, an increase of 64.35% and 69.27% in lipid peroxidation and an increase of 13.07% and 39.68% in protease activity were observed in plants treated with 30% FLL and 30% FFL, respectively. However, treatments with lower concentrations of 5% FLL and 5% FFL exhibited a decline in lipid peroxidation by 2.09% and 2.17% and protease activity by 12.60% and 18.89%, respectively (**Figure 2**). On the other hand, a 30.99% and 76.93% increase in lipid peroxidation and a 62.62% and 50.89% increase in protease activity were reported in wheat plants fed with 10% DLL and 10% DFL leachates of dry parts of marigold (**Figure 3**). More or less similar results were reported in wheat seedlings raised under the laboratory conditions (**Figure 1**) and the older plants raised under field conditions using soil substrates (**Figure 4**) receiving treatments with different concentrations of dry leachates of leaves and flowers of marigold.

### Alterations in antioxidant components of wheat seedlings/plants

**Figure 5:**
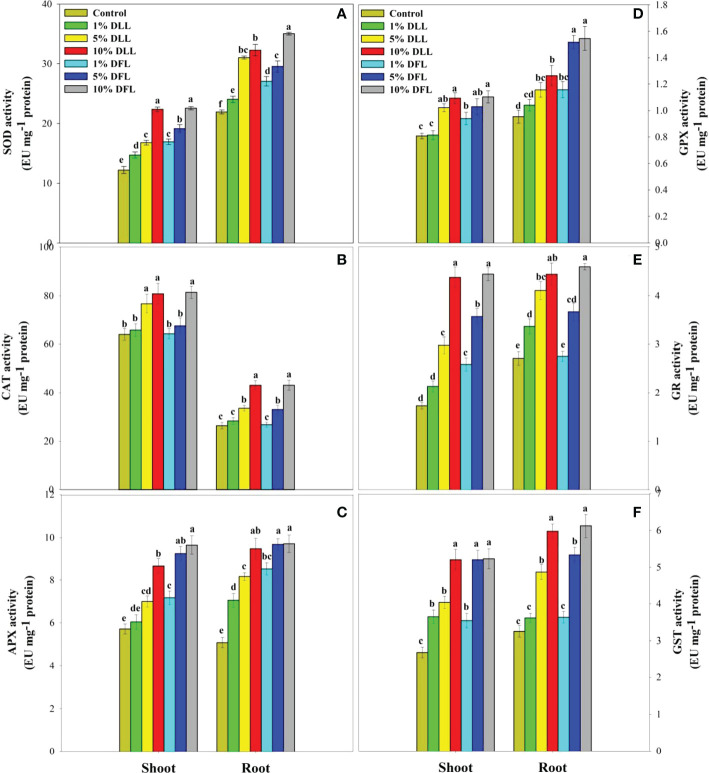
Superoxide dismutase, catalase, ascorbate peroxidase, guaiacol peroxidase, glutathione reductase, and glutathione S-transferase (EU mg^-1^ protein) activity **(A–F)** in *Triticum aestivum* L. seedlings (7 DAS) treated with leachates of dry leaves and flowers of *Tagetes erecta* L. Data followed by the same letters are not significantly different at p< 0.05.

**Figure 6:**
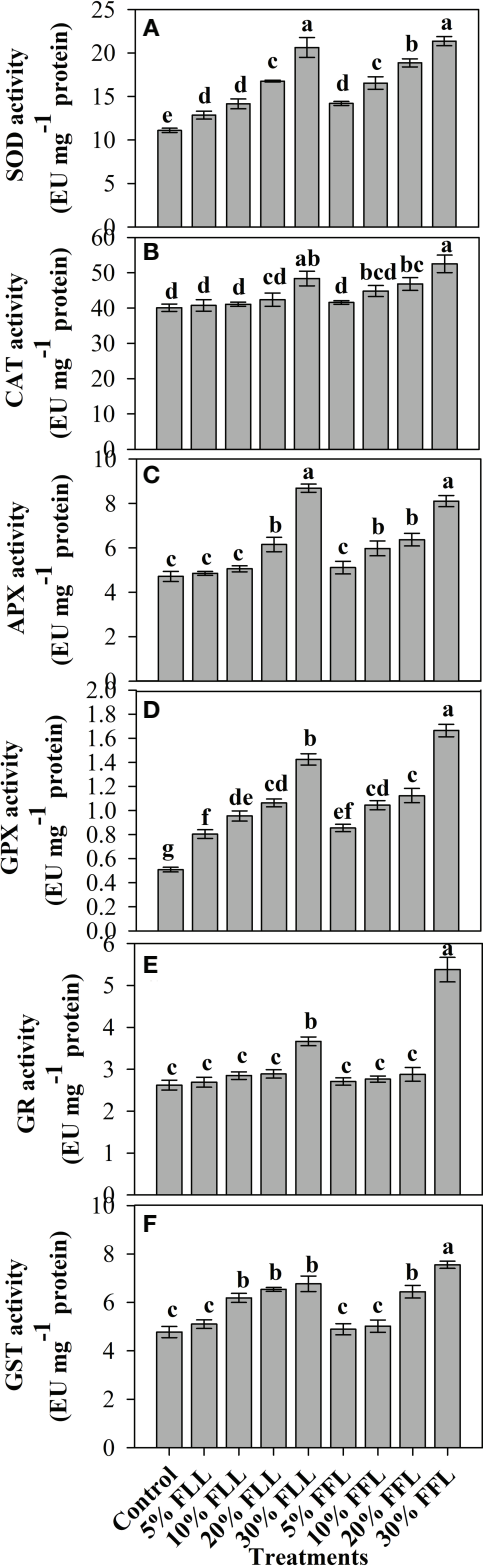
Superoxide dismutase, catalase, ascorbate peroxidase, guaiacol peroxidase, glutathione reductase, and glutathione S-transferase (EU mg^-1^ protein) activity **(A–F)** in leaves of *Triticum aestivum* L. plants raised using sand substrate (25 DAS) treated with leachates of fresh leaves and flowers of *Tagetes erecta* L. Data followed by the same letters are not significantly different at p< 0.05.

**Figure 7:**
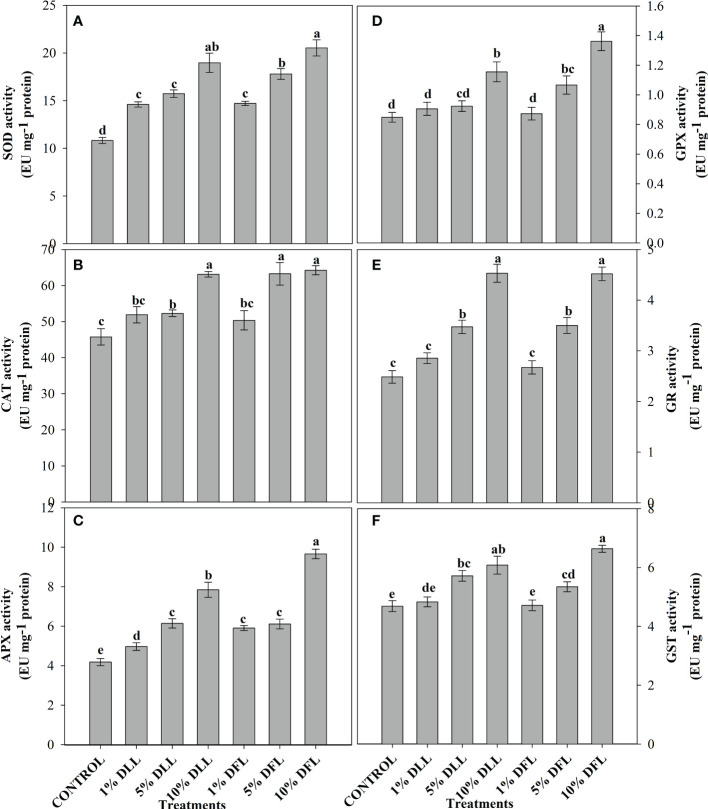
Superoxide dismutase, catalase, ascorbate peroxidase, guaiacol peroxidase, glutathione reductase, and glutathione S-transferase (EU mg^-1^ protein) activity **(A–F)** in leaves of *Triticum aestivum* L. plants raised using sand substrate (25 DAS) treated with leachates of dry leaves and flowers of *Tagetes erecta* L. Data followed by the same letters are not significantly different at p< 0.05.

**Figure 8:**
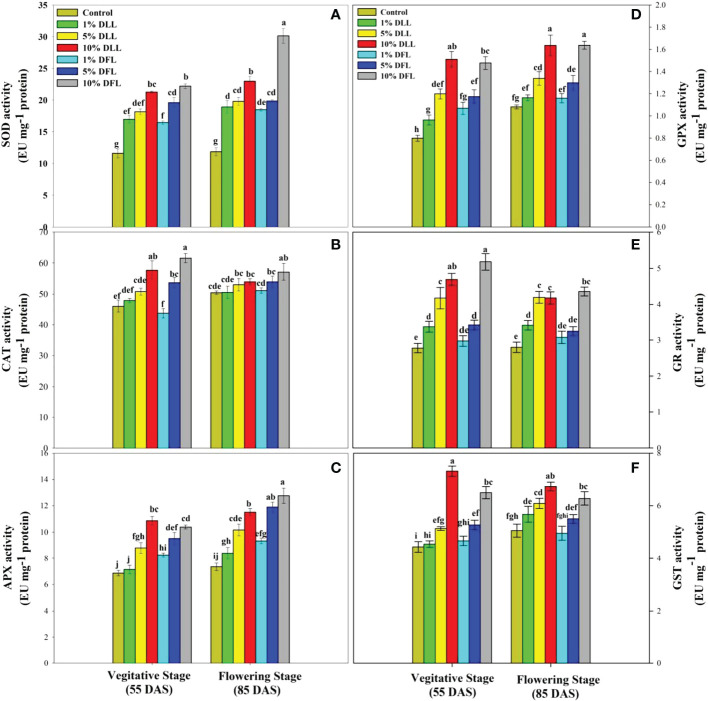
Superoxide dismutase, catalase, ascorbate peroxidase, guaiacol peroxidase, glutathione reductase, and glutathione S-transferase (EU mg^-1^ protein) activity **(A–F)** in flag leaves of *Triticum aestivum* L. raised using soil substrate at different developmental stages treated with leachates of dry leaves and flowers of *Tagetes erecta* L. Data followed by the same letters are not significantly different at p< 0.05.

*Triticum aestivum* L. seedlings/plants subjected to treatments with fresh/dry leaf and flower leachates of marigold showed upregulation of the enzymatic antioxidant defense system. Activities of SOD, CAT, APX, GPX, GR, and GST showed a concentration-dependent increase in wheat seedlings/plants fed with different treatments with leachates, and the maximum increase was noticed when subjected to higher concentrations of fresh (30% w/v) and dry (10% w/v) leachate treatments (**Figures 5**–**8**). In sand culture experiments, SOD, CAT, APX, GPX, GR, and GST contrary to the control counterparts showed an increase of 85.85%, 20.65%, 82.28%, 180.3%, 39.69%, and 41.71% due to 30% FLL treatments and an increase of 92.34%, 31.01%, 72.18%, 225.4%, 104.9%, and 58.28% due to application of 30% FFL leachates of fresh parts, respectively (**Figure 6**). On the other hand, increases of 75.32% and 89.74% in SOD, 37.88% and 40.35% in CAT, 87.79% and 131.1% in APX, 36.47% and 60.0% in GPX, 81.92% and 81.52% in GR, and 29.91% and 41.66% in GST were observed upon application of 10% DLL and 10% DFL leachates of marigold, respectively (**Figure 7**). Treatments with higher concentrations of flower leachates showed more pronounced effects as compared with those of leaf leachates in most of the cases (**Figures 5**–**8**). An almost similar trend was observed in both seedlings raised in laboratory conditions (**Figure 5**) and flag leaves of wheat plants raised in pots (using soil substrate) under field conditions (**Figure 8**) supplemented with different concentrations of leachates of dry leaves and flowers of marigold. Different developmental stages/age of the plant showed significant variation in the activities of SOD, CAT, APX, GPX, GR, and GST, and in most of the cases, maximum activity was recorded at the flowering stage (**Figure 8**).

Glutathione and ascorbic acid contents also exhibited a similar trend (**Tables 1**–**4**) and showed increases of 190.6% and 33.72% by 30% FLL treatments and 211.6% and 45.55% due to 30% FFL leachates of fresh parts (**Table 2**). Increases of 134% and 71.33% were recorded due to 10% DLL treatments and 160% and 82.25% due to 10% DFL leachates of dry parts (**Table 3**). A more or less similar trend was observed in flag leaves of wheat plants at the pre-flowering and flowering stages exposed to 10% DLL and 10% DFL leachates of dry parts of marigold (**Table 4**).

**Table 1:** Reduced glutathione (nM g^-1^ fr wt), ascorbic acid (mg g^-1^ fr wt), phenyl alanine ammonia lyase (nM trans-cinnamic acid min^-1^ mg^-1^ protein), and total antioxidant (percent) activity in *Triticum aestivum* L. seedlings (7 DAS) treated with leachates of dry leaves and flowers of *Tagetes erecta* L.

Treatments	GSH		AsA		PAL activity		DPPH activity	
	SHOOT	ROOT	SHOOT	ROOT	SHOOT	ROOT	SHOOT	ROOT
Control	268.75 ± 11.97^e^	187.50 ± 07.22^e^	1.806 ± 0.027^e^	0.748 ± 0.013^e^	37.75 ± 0.59^d^	23.09 ± 0.55^c^	44.706 ± 0.277^d^	42.914 ± 0.372^c^
1% DLL	343.75 ± 11.96^d^	218.75 ± 11.97^e^	2.059 ± 0.027^c^	0.886 ± 0.022^d^	38.93 ± 0.78^d^	23.37 ± 0.44^c^	45.784 ± 0.404^bc^	43.505 ± 0.150^bc^
5% DLL	393.75 ± 11.97^c^	275.00 ± 10.21^cd^	2.156 ± 0.058^bc^	1.071 ± 0.018^c^	44.03 ± 0.98^b^	25.48 ± 0.55^b^	46.666 ± 0.424^ab^	44.290 ± 0.170^b^
10% DLL	462.50 ± 16.14^b^	356.25 ± 11.96^b^	2.174 ± 0.016^b^	1.578 ± 0.053^a^	49.31 ± 0.67^a^	26.86 ± 0.52^b^	47.549 ± 0.371^a^	44.291 ± 0.161^b^
1% DFL	300.00 ± 10.21^e^	256.25 ± 11.97^d^	1.907 ± 0.012^d^	0.902 ± 0.030^d^	38.51 ± 0.76^d^	23.76 ± 0.61^c^	44.902 ± 0.253^cd^	43.014 ± 0.422^c^
5% DFL	468.75 ± 11.96^b^	300.00 ± 10.20^c^	2.087 ± 0.008^bc^	1.245 ± 0.019^b^	41.22 ± 0.48^c^	25.96 ± 0.63^b^	45.490 ± 0.358^cd^	44.094 ± 0.254^b^
10% DFL	543.75 ± 11.97^a^	425.00 ± 17.68^a^	2.292 ± 0.052^a^	1.561 ± 0.014^a^	42.37 ± 0.54^bc^	28.65 ± 0.69^a^	47.451 ± 0.277^a^	45.371 ± 0.500^a^

Data followed by the same letters are not significantly different at p< 0.05.

**Table 2:** Reduced glutathione (nM g^-1^ fr wt), ascorbic acid, total phenols, tannins (mg g^-1^ fr wt), phenyl alanine ammonia lyase (nM trans-cinnamic acid min^-1^ mg^-1^ protein), and total antioxidant (percent) activity in leaves of *Triticum aestivum* L. plants raised using sand substrate (25 DAS) treated with leachates of fresh leaves and flowers of *Tagetes erecta* L.

Treatments	GSH	AsA	Total Phenols	Tannins	PAL activity	DPPH activity
Control	268.75 ± 11.97^f^	2.239 ± 0.006^g^	1.25 ± 0.01^c^	8.55 ± 0.29^c^	31.45 ± 0.56^f^	55.303 ± 1.275^e^
5% FLL	362.50 ± 16.14^e^	2.246 ± 0.005^fg^	1.29 ± 0.03^bc^	8.58 ± 0.29^c^	31.96 ± 0.22^ef^	58.712 ± 1.294^d^
10% FLL	543.75 ± 18.75^d^	2.306 ± 0.034^fg^	1.38 ± 0.05^bc^	9.63 ± 0.51^c^	32.07 ± 0.37^ef^	62.689 ± 1.251^c^
20% FLL	675.00 ± 27.00^c^	2.410 ± 0.010^e^	1.39 ± 0.04^b^	12.75 ± 0.34^b^	34.10 ± 0.24^cde^	64.205 ± 0.781^bc^
30% FLL	781.25 ± 34.42^ab^	2.994 ± 0.064^b^	1.57 ± 0.06^a^	14.40 ± 0.32^a^	38.97 ± 0.43^ab^	66.856 ± 0.363^b^
5% FFL	587.50 ± 16.14^d^	2.338 ± 0.014^ef^	1.27 ± 0.01^bc^	8.53 ± 0.47^c^	33.30 ± 0.41^def^	58.902 ± 0.947^d^
10% FFL	668.75 ± 21.35^c^	2.518 ± 0.024^d^	1.30 ± 0.02^bc^	9.68 ± 0.53^c^	35.48 ± 1.80^cd^	64.015 ± 0.219^c^
20% FFL	743.75 ± 15.73^b^	2.680 ± 0.049^c^	1.32 ± 0.03^bc^	12.80 ± 0.63^b^	36.45 ± 0.36^bc^	64.962 ± 0.996^bc^
30% FFL	837.50 ± 16.13^a^	3.259 ± 0.038^a^	1.60 ± 0.09^a^	14.98 ± 0.28^a^	40.06 ± 1.58^a^	70.833 ± 0.902^a^

Data followed by same letters are not significantly different at p< 0.05.

**Table 3:** Reduced glutathione (nM g^-1^ fr wt), ascorbic acid, total phenols, tannins (mg g^-1^ fr wt), phenyl alanine ammonia lyase (nM trans-cinnamic acid min^-1^ mg^-1^ protein), and total antioxidant (percent) activity in leaves of *Triticum aestivum* L. plants raised using sand substrate (25 DAS) treated with leachates of dry leaves and flowers of *Tagetes erecta* L.

Treatments	GSH	AsA	Total Phenols	Tannins	PAL activity	DPPH activity
**Control**	312.50 ± 16.14^e^	1.978 ± 0.031^e^	2.07 ± 0.09^a^	11.03 ± 0.11^d^	42.39 ± 0.15^e^	53.205 ± 0.985^c^
1% DLL	431.25 ± 21.35^d^	2.099 ± 0.046^e^	2.10 ± 0.11^a^	11.50 ± 0.28^d^	44.95 ± 0.41^cd^	53.997 ± 0.722^c^
5% DLL	662.50 ± 29.75^c^	2.877 ± 0.039^c^	2.14 ± 0.03^a^	13.45 ± 0.52^c^	48.00 ± 0.22^b^	54.029 ± 0.528^c^
10% DLL	731.25 ± 21.35^b^	3.389 ± 0.034^b^	2.20 ± 0.09^a^	17.85 ± 0.31^a^	51.39 ± 1.16^a^	57.509 ± 0.539^a^
1% DFL	418.75 ± 21.34^d^	2.536 ± 0.074^d^	2.07 ± 0.09^a^	10.70 ± 0.36^d^	42.73 ± 1.20^de^	54.121 ± 0.707^bc^
5% DFL	675.00 ± 22.82^bc^	3.317 ± 0.012^b^	2.17 ± 0.10^a^	12.98 ± 0.36^c^	44.23 ± 0.41^de^	56.410 ± 0.859^ab^
10% DFL	812.50 ± 26.02^a^	3.605 ± 0.125^a^	2.24 ± 0.10^a^	16.58 ± 0.37^b^	47.02 ± 1.19^bc^	58.516 ± 0.782^a^

Data followed by the same letters are not significantly different at p< 0.05.

**Table 4:** Reduced glutathione (nM g^-1^ fr wt), ascorbic acid content (mg g^-1^ fr wt), total phenols, tannins (mg g^-1^ dr wt), phenyl alanine ammonia lyase (nM trans-cinnamic acid min^-1^ mg^-1^ protein), and total antioxidant (percent) activity in flag leaves of *Triticum aestivum* L. raised using soil substrate at different developmental stages treated with leachates of dry leaves and flowers of *Tagetes erecta* L.

	Treatments	GSH	AsA	Total phenols	Tannins	PAL activity	DPPH activity
**55 (DAS)**	**Control**	356.25 ± 18.75^f^	2.923 ± 0.031^g^	7.69 ± 0.16^g^	9.15 ± 0.52^g^	64.98 ± 0.25 ^j^	45.267 ± 0.343^g^
	1% DLL	531.25 ± 15.73^e^	3.260 ± 0.090^f^	8.01 ± 0.32^fg^	11.33 ± 0.57^fg^	71.68 ± 0.69^h^	46.433 ± 0.130^fg^
	5% DLL	743.75 ± 18.75^c^	3.580 ± 0.062^de^	8.72 ± 0.18^e^	11.69 ± 0.57^fg^	74.07 ± 0.87^g^	50.454 ± 0.468^cd^
	10% DLL	800.00 ± 27.00^b^	3.599 ± 0.055^de^	9.60 ± 0.14^bcd^	13.33 ± 0.27^f^	83.82 ± 0.33^c^	53.696 ± 0.594^b^
	1% DFL	518.75 ± 11.97^e^	3.280 ± 0.099^f^	7.61 ± 0.24^g^	10.77 ± 0.46^fg^	67.55 ± 0.17^i^	47.860 ± 0.980^ef^
	5% DFL	631.25 ± 15.72^d^	3.399 ± 0.099^f^	8.63 ± 0.39^ef^	12.04 ± 0.12^fg^	76.54 ± 0.09^f^	48.638 ± 0.225^de^
	10% DFL	825.00 ± 17.67^ab^	3.595 ± 0.021^de^	9.82 ± 0.28^bc^	12.73 ± 0.28^f^	79.36 ± 0.37^e^	51.881 ± 0.722^bc^
**85 (DAS)**	**Control**	337.50 ± 16.14^f^	3.421 ± 0.031^ef^	9.13 ± 0.08^de^	25.70 ± 1.84^e^	67.37 ± 0.39^i^	47.688 ± 0.888^ef^
	1% DLL	562.50 ± 26.02^e^	3.626 ± 0.005^d^	9.63 ± 0.18^bcd^	31.10 ± 1.27^cd^	76.89 ± 0.33^f^	47.774 ± 0.532^ef^
	5% DLL	806.25 ± 27.72^b^	3.675 ± 0.010^d^	10.11 ± 0.12^bc^	32.95 ± 1.44^bc^	77.38 ± 0.29^f^	52.568 ± 1.173^b^
	10% DLL	868.75 ± 21.35^a^	3.896 ± 0.005^bc^	11.14 ± 0.25^a^	35.38 ± 1.60^ab^	85.28 ± 0.33^b^	56.078 ± 0.164^a^
	1% DFL	525.00 ± 10.20^e^	3.721 ± 0.041^cd^	9.47 ± 0.13^cd^	29.80 ± 1.19^d^	71.20 ± 0.56^h^	50.257 ± 0.257^cd^
	5% DFL	662.50 ± 16.13^d^	3.940 ± 0.044^b^	10.18 ± 0.05^b^	33.15 ± 0.83^bc^	82.20 ± 0.38^d^	50.599 ± 0.783^cd^
	10% DFL	862.50 ± 16.14^a^	4.685 ± 0.063^a^	11.64 ± 0.33^a^	36.85 ± 1.18^a^	89.15 ± 0.34^a^	53.425 ± 0.850^b^

Data followed by same letters are not significantly different at p< 0.05.

### Effect on the total phenols and tannins

Total phenols and tannins increased in *Triticum aestivum* L. plants exposed to higher concentrations of treatments, i.e., 10%, 20%, and 30% w/v of fresh parts and 5% and 10% w/v of dry parts of marigold (**Tables 2**–**4**); however, the maximum increase was noticed in wheat plants treated with 30% w/v of fresh parts and 10% w/v of dry parts of marigold. An increase in total phenols and tannins by 25.6% and 68.42% due to 30% FLL treatments and by 28% and 75.20% due to 30% FFL leachates of fresh leaves and flowers of marigold (**Table 2**) and by 6.28% and 61.83% due to 10% DLL treatments and 8.21% and 50.31% due to 10% DFL leachates of dry leaves and flowers of marigold was found (**Table 3**). Contents of total phenols and tannins were more at the flowering stage (85 DAS) as compared with the pre-flowering stage (55 DAS). An increase of 22.01% and 27.49% in total phenols and 37.66% and 43.38% in tannins was observed in wheat plants at the flowering stage exposed to 10% DLL and DFL leachates of dry marigold parts (**Table 4**).

Increased contents of phenols in wheat plants treated with leachates of marigold plant parts may impart greater total antioxidant activity (**Tables 1**–**4**). Leachates of fresh parts of 30% FLL and 30% FFL caused an increase in the activity of total antioxidants (DPPH) by 20.89% and 28.08% relative to the control (**Table 2**) and at the same time by 8.08% and 9.98% when supplied with 10% DLL and 10% DFL treatments (**Table 3**) in the sand culture setup. This improvement of metabolites in wheat plants treated with leaf and flower leachates of marigold was accompanied by an increase in activity of (PAL) phenylalanine ammonia lyase (**Tables 1**–**4**). PAL activity increased by 23.91% and 27.37% in wheat plants due to application of 30% FLL and 30% FFL treatments (**Table 2**) and by 21.23% and 10.92% when exposed to 10% DLL and 10% DFL treatments (**Table 3**). Total antioxidant activity and the activity of phenylalanine ammonia lyase showed a more or less similar trend in wheat seedlings/plants raised in laboratory conditions and pots (using soil substrate) under field conditions (**Tables 1**, **4**).

### Accumulation of osmotic constituents in wheat plants

Free sugars in wheat leaves treated with fresh leachates of leaves and flowers of *T. erecta* L. showed little variation (**Table 5**); however, wheat plants fed with higher concentrations, i.e., 10% DLL and 10% DFL of dry parts *T. erecta* L., showed an increase in free sugars by 22.65% and 17.67% (**Table 6**). Nevertheless, an increase in free sugars by 26.82% and 34.13% was also found in flag leaves of 85 DAS-old wheat plants fed with 10% DLL and 10% DFL of dry parts (**Table 7**). Free amino acids and free proline contents in wheat plants raised in sand cultures registered a maximum increase by 17% and 113.4% up on application of 30% FLL treatments and by 34.01% and 92.32% due to application of 30% FFL leachates of fresh parts (**Table 5**). On the other hand, the enhancement in free amino acids and free proline contents in wheat plants was 243.5% and 46.77% when exposed to 10% DLL treatments and 297% and 44.41% when exposed to 10% DFL leachates of dry parts of *Tagetes erecta* L. (**Table 6**). Wheat plants raised in pots using soil substrates under a field setup also showed a similar trend (**Table 7**).

**Table 5:** Free sugars, free amino acids (mg g^-1^ fr wt), and free proline (μ mole g^-1^ fr wt) contents in leaves of *Triticum aestivum* L. plants raised using sand substrate (25 DAS) treated with leachates of fresh leaves and flowers of *Tagetes erecta* L.

Treatments	Free sugars	Free amino acids	Free proline
Control	1.26 ± 0.04^d^	7.35 ± 0.31^cd^	9.40 ± 0.65^d^
5% FLL	1.30 ± 0.03^d^	6.91 ± 0.29^d^	11.04 ± 1.28^d^
10% FLL	1.31 ± 0.02^d^	6.75 ± 0.30^d^	16.56 ± 1.64^bc^
20% FLL	1.33 ± 0.03^cd^	8.12 ± 0.32^bc^	17.88 ± 1.35^ab^
30% FLL	1.36 ± 0.01^cd^	8.60 ± 0.14^b^	20.06 ± 0.24^a^
5% FFL	1.32 ± 0.04^cd^	6.51 ± 0.33^d^	14.61 ± 0.72^c^
10% FFL	1.44 ± 0.03^bc^	8.04 ± 0.31^bc^	15.31 ± 1.01^bc^
20% FFL	1.53 ± 0.03^b^	8.64 ± 0.30^b^	17.22 ± 0.33^bc^
30% FFL	1.96 ± 0.09^a^	9.85 ± 0.44^a^	18.07 ± 0.31^ab^

Data followed by the same letters are not significantly different at p<0.05.

**Table 6:** Free sugars, free amino acids (mg g^-1^ fr wt) and free proline (μ mol g^-1^ fr wt) contents in leaves of *Triticum aestivum* L. plants raised using sand substrate (25 DAS) treated with leachates of dry leaves and flowers of *Tagetes erecta* L.

Treatments	Free sugars	Free amino acids	Free proline
**Control**	1.81 ± 0.02^c^	3.10 ± 0.12^e^	28.82 ± 0.96^e^
1% DLL	1.92 ± 0.08^bc^	3.66 ± 0.32^de^	35.72 ± 0.29^bc^
5% DLL	1.83 ± 0.04^c^	4.29 ± 0.19^d^	38.10 ± 0.10^ab^
10% DLL	2.22 ± 0.06^a^	10.65 ± 0.68^b^	42.30 ± 1.42^a^
1% DFL	1.50 ± 0.13^d^	4.15 ± 0.07^d^	27.51 ± 1.43^de^
5% DFL	2.03 ± 0.16^abc^	5.66 ± 0.30^c^	31.51 ± 0.78^cd^
10% DFL	2.13 ± 0.12^ab^	12.31 ± 0.42^a^	41.62 ± 2.01^a^

Data followed by same letters are not significantly different at p< 0.05.

**Table 7:** Free sugars, free amino acids (mg g^-1^ dr wt), and free proline (μ mole g^-1^ dr wt) contents in flag leaves of *Triticum aestivum* L. raised using soil substrate at different developmental stages treated with leachates of dry leaves and flowers of *Tagetes erecta* L.

	Treatments	Free sugars	Free amino acids	Free proline
**55 (DAS)**	**Control**	5.76 ± 0.19^g^	10.05 ± 0.26^h^	178.37 ± 3.72^g^
	1% DLL	5.75 ± 0.19^g^	12.00 ± 0.10^g^	179.47 ± 0.88^g^
	5% DLL	6.56 ± 0.05^f^	14.99 ± 0.19^f^	187.37 ± 1.75^ef^
	10% DLL	6.68 ± 0.05^f^	17.46 ± 0.53^e^	191.59 ± 3.07^de^
	1% DFL	5.85 ± 0.16^g^	11.06 ± 0.27^gh^	178.63 ± 2.33^g^
	5% DFL	6.65 ± 0.21^f^	14.85 ± 0.26^f^	182.63 ± 2.48^fg^
	10% DFL	7.28 ± 0.05^e^	16.93 ± 0.10^e^	195.08 ± 5.44^d^
**85 (DAS)**	**Control**	8.76 ± 0.21^d^	30.21 ± 0.43^d^	203.19 ± 3.45^c^
	1% DLL	10.14 ± 0.06^c^	31.71 ± 0.58^cd^	208.34 ± 1.01^c^
	5% DLL	10.85 ± 0.10^b^	32.67 ± 0.54^c^	217.67 ± 0.79^a^
	10% DLL	11.11 ± 0.12^b^	38.75 ± 0.81^a^	219.38 ± 0.27^a^
	1% DFL	9.18 ± 0.42^d^	32.00 ± 0.91^cd^	209.56 ± 0.68^bc^
	5% DFL	10.11 ± 0.30^c^	33.50 ± 1.70^c^	216.53 ± 1.31^ab^
	10% DFL	11.75 ± 0.32^a^	36.15 ± 0.42^b^	219.46 ± 0.98^a^

Data followed by the same letters are not significantly different at p< 0.05.

### Variation in relative water content, length, biomass, and yield attributes in wheat plants

RWC (relative water content) decreases in wheat plants with the increase in the concentration of leachates, and a maximum decline was observed in wheat plants treated with 10% DLL and 10% DFL leachates of dry parts. Plant height, spike length, number of spikelets per spike, and 100 grain weight of the wheat plants registered an increase of 5.94%, 14.25%, 30.30%, and 3.49% due to application of 1% DLL and of 6.42%, 20.21%, 33.33%, and 4.19% due to 1% DFL leachate treatments, whereas a reduction in plant height by 7.39% and 5.83%, spike length by 5.74% and 4.89%, number of spikelets per spike by 27.27% and 33.33%, and 100 grain weight by 3.84% and 3.14% was recorded due to application of 10% DLL and 10% DFL leachates of dry leaves and flowers of *Tagetes erecta* L. (**Table S1**).

## Discussion

Allelopathic effects on plant species are directly reflected in altered physiology and biochemistry. Both inhibitory and stimulatory modulations do have a direct relationship with the cellular structural and functional stability. In the present study, it was observed that *Triticum aestivum* L. seedlings/plants showed significant enhancement in lipid peroxidation when exposed to higher concentrations of fresh/dry leachates of marigold leaves and flowers. Stress imposition exhibited increased generation of H_2_O_2_ imparting greater peroxidation of lipids, which results in leakage of important cellular components (Sofo et al., 2004). Zhang et al. (2007), while working on mangrove plants, noticed that the lipid peroxidation level in leaves may serve as a biomarker in *Bruguiera gymnorrhiza* whereas POD activity may be used as heavy metal biomarker for *Kandelia candel*. The enhancement in lipid peroxidation has also been reported in different plants facing extreme environmental conditions like salt stress (Borzouei et al., 2012; Ahanger et al., 2020), stress imposed by heavy metals (Singh et al., 2017), and allelopathic stress as well (Al-Hawas and Azooz, 2018; da Silva and Vieira, 2019). In addition, the oxidative effects of leachates were also evident from the altered protease activity reflecting in greater damage to protein structures and hence to their functioning. It was evident that exposure to higher concentrations of leachates resulted in increased protease activity in both laboratory and pot experiments (**Figures 1B**, **2B**, **3B**, **4B**). Plant proteases play an important role in many different biological processes including stomatal development and distribution, as well as systemic stress response. They are also related to the increase in ROS production detected during plant exposure and response to environmental stress (Mangena, 2022). Proteases mitigate this process by degradation of damaged, denatured proteins, remobilizing amino acids and generating molecules involved in signal transduction (D’Ippólito et al., 2021). The upregulation of protease activity in plants facing extreme environments has been reported earlier as well (Ahanger et al., 2020; Tittal et al., 2020).

An increase of total phenols and tannins was observed in *Triticum aestivum* L. at different developmental stages up on application of leachates of leaves and flowers of *Tagetes erecta* L., which may be due to the presence of allelochemicals in these leachates. The metabolite accumulation increases with the increase in the concentration of leachates. Increased contents of phenols in treated wheat plants may impart greater total antioxidant activity, and this improvement of metabolites in wheat plants was accompanied by an increase in activity of phenylalanine ammonia lyase (PAL). This increase in the antioxidant components in wheat plants in response to treatments of leaf and flower leachates of marigold indicated stress imposition by allelochemicals present in these leachates of plant parts. Plants exposed to a variety of environmental stresses results in oxidative stress; the uncontrolled production and accumulation of ROS is one of the effects of allelochemicals on target plants which affect biological processes triggering changes in cellular metabolism accompanied by activation of the antioxidant defense system. The inappropriate balance between ROS production and the counterbalance of free radicals by the antioxidant defense system causes damage to the cellular components which may lead to cell death. Antioxidants’ components are important for the maintenance of ROS at the physiological level (Xie et al., 2019) maintaining cellular homeostasis (Gill and Tuteja, 2010; Das and Roychoudhury, 2014) by scavenging ROS and also preventing the formation of toxic radicals.

The activities of antioxidant enzymes such as SOD, CAT, APX, GR, and GPX along with the non-enzymatic antioxidant components such as AsA and GSH were considerably affected in wheat seedlings when exposed to shoot extracts of *Achillea santolina* (Hatata and El-Darier, 2009). In the present study also, it was observed that activity of enzymatic antioxidants and the contents of non-enzymatic ones showed an apparent increase reflecting the potential to induce the counteractive mechanisms for lessening the allelopathic effects. Increased peroxidation of lipids and alterations in antioxidant enzymes in different plants upon exposure to allelochemical stress have been reported earlier as well (Cruz-Ortega et al., 2007; Tomar et al., 2015). The concentration-dependent enhancement in the activity of enzymatic antioxidants like superoxide dismutase, catalase, and peroxidase has been reported in *Brassica oleracea* seedlings when subjected to the treatment of *Calotropis procera* extracts (Gulzar and Siddiqui, 2017) and in pea seedlings also when exposed to *Artemisia monosperma* and *Thymus vulgaris* extracts (Al-Hawas and Azooz, 2018). *Sesamum indicum* L. has also shown a significant increase in the activity of antioxidant enzymes up on exposure to sorghum aqueous leaf and stem extract (Murimwa et al., 2019).

In the present study, it was also noticed that osmotic components/osmolytes including sugars, proline, and free amino acids increased in wheat seedlings grown with leachate treatments. Osmolytes are low-molecular-weight organic solutes, the biosynthesis of which is influenced by the environment. Osmolytes protect subcellular structures from ROS and contribute toward turgor maintenance and osmotic adjustments (Slama et al., 2015). The accumulation of free proline has also been reported in lettuce seedlings subjected to different stages of shoot aqueous extracts of diploid and mixoploid fenugreek (Omezzine et al., 2014). Singh et al. (2006) have reported the accumulation of free proline in *Cassia occidentalis* as a result of treatment of α-pinene. These osmoregulators like sugars and amino acids play a significant part in stabilization of cells and tissues of plants particularly under harsh conditions by adjusting the osmotic pressure (Hasegawa et al., 2000). Accumulation of soluble sugars increased in *Zea mays* by the allelopathic potential of aqueous extract of air-dried olive processing waste in a concentration-dependent way (Saleh, 2013). Aqueous extract of roots and leaves of *Heliotropium bacciferum* Forssk gradually increased the proline and soluble sugars in *Oryza sativa* and *Teucrium polium* plants (Al-Taisan, 2014), and in this respect leaves of *Heliotropium bacciferum* were more effective than roots. An increased amount of proline and soluble sugars served as a defense mechanism in the growth of *Raphanus sativus* L. against the allelochemical stress induced by water extracts of aerial parts of peppermint having phenolic compounds like trans-ferulic acid (10.8 mg/g), hesperidin (9.3 mg/g), ellagic acid (6.8 mg/g), and sinapic acid (4.2 mg/g) detected using HPLC (Mahdavikia et al., 2017). Unal et al. (2017) have reported an increase in proline content in wild mustard plants against the allelopathic effects of *Palustriella falcata*. An enhancement of proline has also been reported in *Phaseolus vulgaris* seedlings against allelopathic effects of an alkaloid fraction of *Crotalaria retusa* Linn (Ogunsusi et al., 2018).

RWC (relative water content), height, spike length, number of spikelets per spike, and 100 grain weight of the wheat plants raised with the leachates of dry leaves and flowers of *Tagetes erecta* L. exhibited a slight increase at lower concentrations of 1% w/v, whereas a decrease was recorded at higher concentrations of 10% w/v. A decrease in relative water content has been reported earlier in *Brassica campestris* L. (mustard) treated with aqueous extracts of the roots, leaves, and seeds of *Cassia tora* L. by Sarkar et al. (2012). Similarly, a decrease in RWC was also reported in *Chenopodium album* L., *Melilotus alba*, and *Nicotiana plumbaginifolia* plants subjected to allelopathic stress imposed by *Cassia sophera* L. extracts (Gulzar et al., 2014) and in *Brassica oleracea* plants exposed to *Calotropis procera* extracts (Gulzar and Siddiqui, 2017).

A decline in length, biomass, and yield attributes was also observed in *Oryza sativa* L. and *Triticum aestivum* L. plants in response to incorporation of sunflower residues (Bashir et al., 2012). A reduction in growth of lettuce was registered when exposed to shoot aqueous extracts of diploid and mixoploid fenugreek (Omezzine et al., 2014). A decrease in germination, length, and biomass accumulation has been observed in *Chenopodium album* L., *Melilotus alba*, and *Nicotiana plumbaginifolia* plants in response to *Cassia sophera* L. extracts (Gulzar et al., 2014), in wheat plants in response to *Jatropha curcas* leachates/extracts (Tomar et al., 2015), in *Brassica oleracea* plants in response to *Calotropis procera* extracts (Gulzar and Siddiqui, 2017), and in *Eleusine coracana* in response to higher concentrations of leaf leachate extracts of (mangrove) *Excoecaria agallocha* L. The inhibition can be related to the allelochemicals like flavonoids and phenolic acids (Desai et al., 2017).

## Conclusion

The above results indicate that the leachates of fresh/dry parts of *Tagetes erecta* L. at higher concentrations enhance the antioxidant and osmotic components of wheat (*Triticum aestivum* L.) seedlings/plants, which probably reflects on their growth and yield parameters. Treatments of dry leachates have greater impact than fresh leachates, and the effect of flower leachates in general was more than that of leaf leachates. The leachate treatments show inhibitory effects on the growth and yield of wheat at higher concentrations only, probably indicating that continuous/mismanaged decomposition of *Tagetes erecta* L. in the agriculture field may need monitoring. Moreover, understanding the mechanisms of possible allelochemical action may impart a basis for the development of new natural biodegradable herbicides to control weeds maintaining an ecofriendly environment for crops in order to boost production in sustainable agriculture; however, further experiments are needed in this context.

## Data availability statement

The raw data supporting the conclusions of this article will be made available by the authors, without undue reservation.

## Author contributions

This manuscript contains part of the PhD work of RM. This study was conceived and designed by Prof. RA. RM performed the experiments and wrote the first draft of the manuscript, and RA cross-checked the results and manuscript. SA and MA helped in the experiments, and KJ helped in the literature survey. All authors contributed to the article and approved the submitted version.

## Acknowledgments

The authors are thankful to Head, School of Studies in Botany, Jiwaji University, Gwalior, for providing necessary facilities.

## Conflict of interest

The authors declare that the research was conducted in the absence of any commercial or financial relationships that could be construed as a potential conflict of interest.

## Publisher’s note

All claims expressed in this article are solely those of the authors and do not necessarily represent those of their affiliated organizations, or those of the publisher, the editors and the reviewers. Any product that may be evaluated in this article, or claim that may be made by its manufacturer, is not guaranteed or endorsed by the publisher.
