# Supplementary material for: Differential activity of wheat antioxidant defense system and alterations in the accumulation of osmolytes at different developmental stages as influenced by marigold (Tagetes erecta L.) leachates
Source: Front Plant Sci. 2022 Dec 1;13:1001394. doi: 10.3389/fpls.2022.1001394 (PMC9751799; doi:10.3389/fpls.2022.1001394)
Supplement: Supplementary file 1 [file Table_1.docx]

**Table S1:** RWC, Height, spike length, number of spikelets per spike and 100 grain weight of *Triticum aestivum* L. raised using soil substrate treated with leachates of dry leaves and flowers of *Tagetes erecta* L. at flowering stage. Data followed by same letters are not significantly different at p<0.05.

| **Treatments** | **RWC (%)** | **Plant Hight (cm)** | **Spike Length (cm)** | **Number of spikelets per spike** | **100 grain Weight (g)** |
| --- | --- | --- | --- | --- | --- |
| Control | 87.19±0.52^a^ | 52.46±2.55^ab^ | 4.70±0.21^b^ | 8.25±0.25^b^ | 2.86±0.05^abc^ |
| 1% DLL | 89.24±0.52^a^ | 55.58±2.82^a^ | 5.37±0.24^a^ | 10.75±0.47^a^ | 2.96±0.08^ab^ |
| 5% DLL | 86.31±0.60^a^ | 50.51±2.14^ab^ | 4.67±0.23^b^ | 8.25±0.25^b^ | 2.81±0.03^c^ |
| 10% DLL | 85.94±0.84^a^ | 48.58±2.45^b^ | 4.43±0.18^b^ | 6.00±0.40^c^ | 2.75±0.03^c^ |
| 1% DFL | 88.02±1.84^a^ | 55.83±2.19^a^ | 5.65±0.16^a^ | 11.00±0.41^a^ | 2.98±0.07^a^ |
| 5% DFL | 86.49±3.99^a^ | 52.17±2.25^ab^ | 4.75±0.21^b^ | 7.75±0.25^b^ | 2.82±0.02^bc^ |
| 10% DFL | 85.61±3.06^a^ | 49.40±1.79^ab^ | 4.47±0.23^b^ | 5.50±0.29^c^ | 2.77±0.02^c^ |
